# Nitrogen-Fixing Trees Shift Soil Organic Carbon Toward Mineral-Associated Stability by Enhancing Microbial-Derived Carbon in Subtropical Plantations

**DOI:** 10.3390/plants15152367

**Published:** 2026-07-31

**Authors:** Hongwei Bai, Yinying He, Qing Liu, Junhao Chen, Wending Tan, Mei Yang, Xianyu Yao

**Affiliations:** Guangxi Colleges and Universities Key Laboratory for Cultivation and Utilization of Subtropical Forest Plantation, School of Forestry, Guangxi University, Nanning 530004, China; shimei33zishi@163.com (H.B.); 18778228693@163.com (Y.H.); 19017098024@163.com (Q.L.); junhaochen110128@163.com (J.C.); twd2051172080@163.com (W.T.)

**Keywords:** soil organic carbon fractions, south subtropical plantation, N-fixing tree species, microbial community, extracellular enzymes

## Abstract

Introducing nitrogen (N)-fixing trees into plantations is a promising strategy to increase soil organic carbon (SOC) storage. However, whether this practice enhances SOC stability, a key determinant of long-term carbon sequestration, and through which specific microbial pathways, remains poorly understood. Here, we compared pure Eucalyptus plantations with mixed plantations containing the N-fixing tree *Erythrophleum fordii* (*E. fordii*) in subtropical China by measuring SOC fractions, microbial communities, extracellular enzymes, and microbial-derived carbon (MNC) content. After five years of plantation establishment, mixed plantations significantly increased total SOC by 22.41% compared to pure eucalypt stands. More importantly, this increase was accompanied by a pronounced shift in the carbon pool toward more stable mineral-associated organic carbon (MAOC), which increased by 28.39% in the surface layer. This increase was associated with microbial community restructuring characterized by a greater fungal contribution. The fungal-derived carbon (FNC) rose by 51.20%, and structural equation modeling showed that MNC contributed directly to MAOC formation, a stronger effect than the indirect enzyme-mediated pathway. Our findings provide field-based evidence that mixing *Eucalyptus* with N-fixing trees enhances SOC stability, primarily through the association between FNC accumulation and MAOC formation. This microbial mechanism provides a process-based perspective for evaluating mixed-species N-fixing tree plantations as a carbon-friendly management strategy in subtropical forestry.

## 1. Introduction

Forest soil is an important carbon pool and offers a natural solution to address climate change [1,2]. Expanding forest area and enhancing the forest C sink have been identified as a key strategy in climate mitigation plans worldwide. Plantation forests, in particular, have expanded rapidly and play an increasingly significant role in global C cycling [3]. However, the successive planting of monoculture plantations, especially short-rotation industrial forests, can reduce soil organic carbon (SOC) sequestration [4]. Developing mixed plantations with N-fixing tree species has emerged as a promising silvicultural option to enhance SOC storage [5,6], as N-fixing trees can improve soil biological, chemical, and physical properties, thereby influencing biogeochemical cycles and ecosystem productivity. Considerable attention has been devoted to examining the effects of N-fixing tree species on above-ground growth and the cycling of soil nitrogen and phosphorus. Leguminous N-fixing trees can establish symbiotic associations with rhizobial microorganisms, allowing biological nitrogen fixation and additional nitrogen inputs into forest ecosystems. *Erythrophleum fordii* (*E. fordii*) is a native leguminous tree species commonly used in subtropical restoration and mixed plantation systems, and its capacity to associate with nitrogen-fixing microorganisms provides a potential mechanism for improving soil nitrogen availability. However, the magnitude of biological nitrogen fixation contribution and its relative importance compared with litter inputs, root turnover, and rhizosphere processes remain insufficiently quantified in subtropical plantations. Although N-fixing tree species strongly impact SOC sequestration [6], there is a paucity of information regarding the pathways and mechanisms through which they influence soil C retention, highlighting an area in need of urgent research [7,8].

The impact of N-fixing trees on the soil C pool is intricate, with microbial processes being a key potential pathway [6,7,8]. Many N-fixing tree species produce relatively N-rich litter [9], and *E. fordii* has been shown to have a low C:N ratio [10], which increases soil nitrogen availability upon decomposition This input of high-quality litter and subsequent nitrogen enrichment can alter soil microbial community composition and the activity of extracellular enzymes involved in C and N cycling [11,12,13]. In particular, nitrogen enrichment may alter microbial community structure toward greater fungal contribution and enhance the activities of key enzymes such as β-1,4-glucosidase (BG) and N-acetyl-β-glucosaminidase (NAG) [14]. These changes in microbial attributes are closely linked to the accumulation of microbial-derived carbon (MNC), which has been recognized as a crucial precursor for the formation of stable SOC [15,16].

Not all SOC fractions contribute equally to long-term C sequestration. Particulate organic carbon (POC) is physically unprotected, derives primarily from partially decomposed plant debris, and turns over in years to decades. In contrast, mineral-associated organic carbon (MAOC) is stabilized through chemical bonding to soil minerals (e.g., iron and aluminum oxides, clay minerals) and persists for decades to centuries [17,18]. The Microbial Efficiency-Matrix Stabilization (MEMS) framework provides a theoretical basis for understanding how litter quality influences this partitioning. High-quality litter with a low C:N ratio is efficiently metabolized by microorganisms [19,20], and the resulting MNC has a high affinity for mineral surfaces. This MNC thus becomes preferentially incorporated into the MAOC pool. Low-quality litter with a high C:N ratio, by contrast, tends to persist as partially decomposed fragments and contributes primarily to the POC pool. This framework suggests that the low Litter C:N from N-fixing trees may enhance MAOC formation through a microbial-derived pathway, implying that N-fixing species increase stable soil C components of microbial origin [6,21]. However, empirical evidence from field plantations directly linking changes in litter quality, microbial community composition (e.g., the fungal-to-bacterial ratio), enzyme activities, and MNC accumulation to shifts in SOC physical fractions (POC vs. MAOC) remains limited [22,23]. Recent studies have highlighted the potential role of fungal-derived carbon (FNC) in MAOC formation due to its chemical recalcitrance and mineral affinity [16,24].

Here, we addressed these questions by conducting a field experiment in subtropical plantations. We compared pure Eucalyptus plantations (PP) with mixed plantations containing the N-fixing tree *Erythrophleum fordii* (MP). We measured Litter C:N ratio, soil nitrogen availability, microbial community composition, enzyme activities, microbial-derived C (MNC), and SOC physical fractions. We hypothesized that (i) the lower C:N litter input from N-fixing trees would increase soil nitrogen availability, thereby altering microbial community structure toward greater fungal contribution and enhancing enzyme activities, and (ii) this microbial restructuring would promote (MNC) accumulation, and FNC in particular would contribute preferentially to MAOC over POC due to its greater mineral affinity [16,24], resulting in a higher MAOC:SOC ratio in mixed plantations.

## 2. Results

### 2.1. Differences in Litter and Soil Physicochemical Properties Between PP and MP

Except for the non-significant impact on Litter C, the mixed plantation with N-fixing trees significantly affected Litter N contents and their C:N ratio (Table 1). Compared with PP, MP had significantly higher Litter N by 35.12%, respectively, while Litter C:N was significantly lower by 25.07%. Consistent with the decrease in Litter C:N, soil C:N in the 0–20 cm layer of MP was significantly lower than that of PP by 18.19% (Table 2). Both stand type and soil depth had significant effects on pH, TN, nitrate nitrogen (NO_3_^−^-N), ammonium nitrogen (NH_4_^+^-N), and soil C:N (*p* < 0.05), but did not significantly affect total phosphorus (TP). Their interaction only significantly affected NO_3_^−^-N (*p* < 0.05). In the 0–20 cm layer, MP had significantly higher total nitrogen (TN) and NO_3_^−^-N than PP by 48.13% and 46.14%, respectively, and significantly lower pH by 10.60%. In the 20–40 cm layer, TN in MP was significantly higher by 36.64% compared with PP, and pH was significantly lower by 9.56%. Overall, in both soil layers, the mixed plantation tended to have higher soil N content and lower pH than the pure plantation.

### 2.2. Differences in SOC Fractions and MNC Between PP and MP

**Figure 1:**
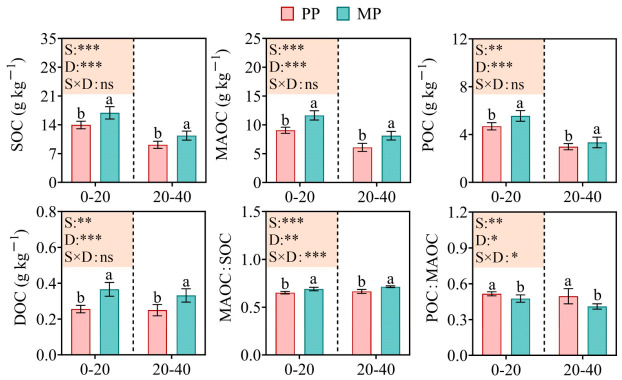
Differences in SOC components between PP and MP. Values are presented as means with standard deviations (*n* = 5). Different letters (a, b) above the bars indicate the significant differences (ANOVA, SPSS v26.0, https://www.ibm.com/products/spss-statistics (accessed on 10 May 2026), *p* < 0.05). PP: Pure Eucalyptus plantation; MP: *Eucalyptus*/*Erythrophleum fordii* (*E. fordii*) *plantation*; S: stand type, D: soil depth; S × D: interaction effect between stand type and soil depth (*, **, and *** indicate significant differences at *p* < 0.05, *p* < 0.01, and *p* < 0.001, ns indicate *p* > 0.05).

Both stand type and soil depth significantly affected SOC and its fractions, including Dissolved Organic Carbon (DOC), (*p* < 0.05), whereas their interaction did not significantly influence the contents of SOC or its fractions (Figure 1). Specifically, the MP stand type exhibited significantly higher contents of SOC, MAOC, POC, and DOC. In the 0–20 cm layer, SOC, MAOC, POC, and DOC in MP were higher than those in PP by 21.17%, 28.39%, 18.35%, and 43.51%, respectively. In the 20–40 cm soil layer, SOC, MAOC, and DOC in MP were higher than those in PP by 24.29%, 33.40%, and 32.08%, respectively, while no significant difference in POC content was observed between MP and PP in this layer (*p* > 0.05). The introduction of N-fixing trees shifted the SOC pool towards more stable carbon forms (Figure 1). Specifically, in both soil layers, the MAOC:SOC ratio was significantly higher in MP compared to PP, whereas the POC:MAOC ratio was significantly lower in MP than in PP (*p* < 0.05).

**Figure 2:**
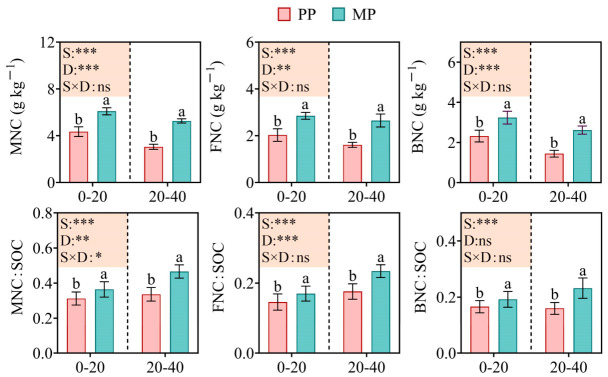
Differences in microbial-derived carbon components between PP and MP. Values are presented as means with standard deviations (*n* = 5). Different letters (a, b) above the bars indicate the significant differences (ANOVA, SPSS, *p* < 0.05). PP: Pure Eucalyptus plantation; MP: *Eucalyptus*/*E. fordii plantation*; S: stand type, D: soil depth; S × D: interaction effect between stand type and soil depth (*, **, and *** indicate significant differences at *p* < 0.05, *p* < 0.01, and *p* < 0.001, ns indicate *p* > 0.05).

**Figure 3:**
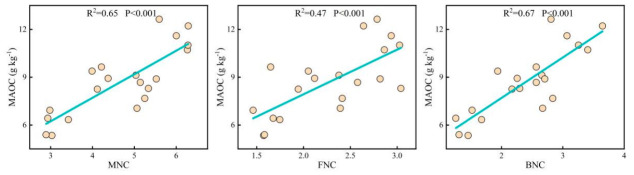
Linear correlation plots between soil MNC, FNC, BNC, and MAOC. MAOC: mineral-associated organic carbon; MNC: microbial-derived carbon; FNC: funga-derived carbon; BNC: bacterial-derived carbon.

Both stand type and soil depth significantly influenced MNC and its components, whereas their interaction (S × D) significantly affected only MNC:SOC (Figure 2), with no significant effects on other parameters (*p* > 0.05). Meanwhile, in both the 0–20 cm and 20–40 cm layers, MNC, FNC, and bacterial-derived carbon (BNC) in MP were significantly higher than those in PP. However, the ratios MNC:SOC, FNC:SOC, and BNC:SOC showed significant advantages in MP only in the 20–40 cm layer, with no significant differences between MP and PP in the 0–20 cm layer (*p* > 0.05). Linear correlation analysis further revealed that MAOC content was significantly positively correlated with MNC, FNC, and BNC (Figure 3; *p* < 0.001).

### 2.3. Differences in Bacterial and Fungal Biomass and Soil Extracellular Enzyme Activities Between PP and MP

**Figure 4:**
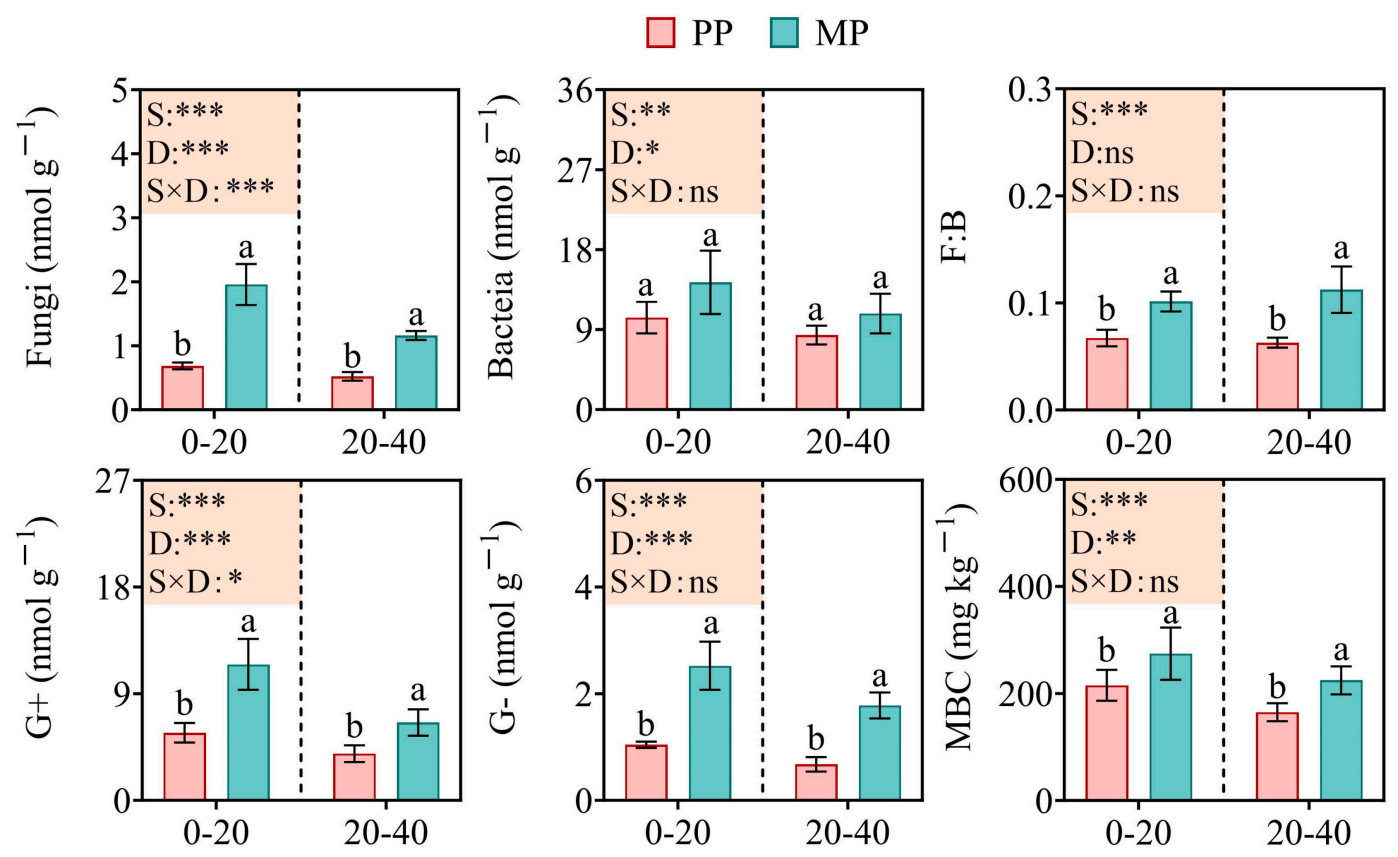
Differences in soil microorganisms between PP and MP. Values are presented as means with standard deviations (*n* = 5). Different letters (a, b) above the bars indicate the significant differences (ANOVA, SPSS, *p* < 0.05). PP: Pure Eucalyptus plantation; MP: *Eucalyptus*/*E. fordii plantation*; S: stand type, D: soil depth; S × D: interaction effect between stand type and soil depth (*, **, and *** indicate significant differences at *p* < 0.05, *p* < 0.01, and *p* < 0.001, ns indicate *p* > 0.05).

**Figure 5:**
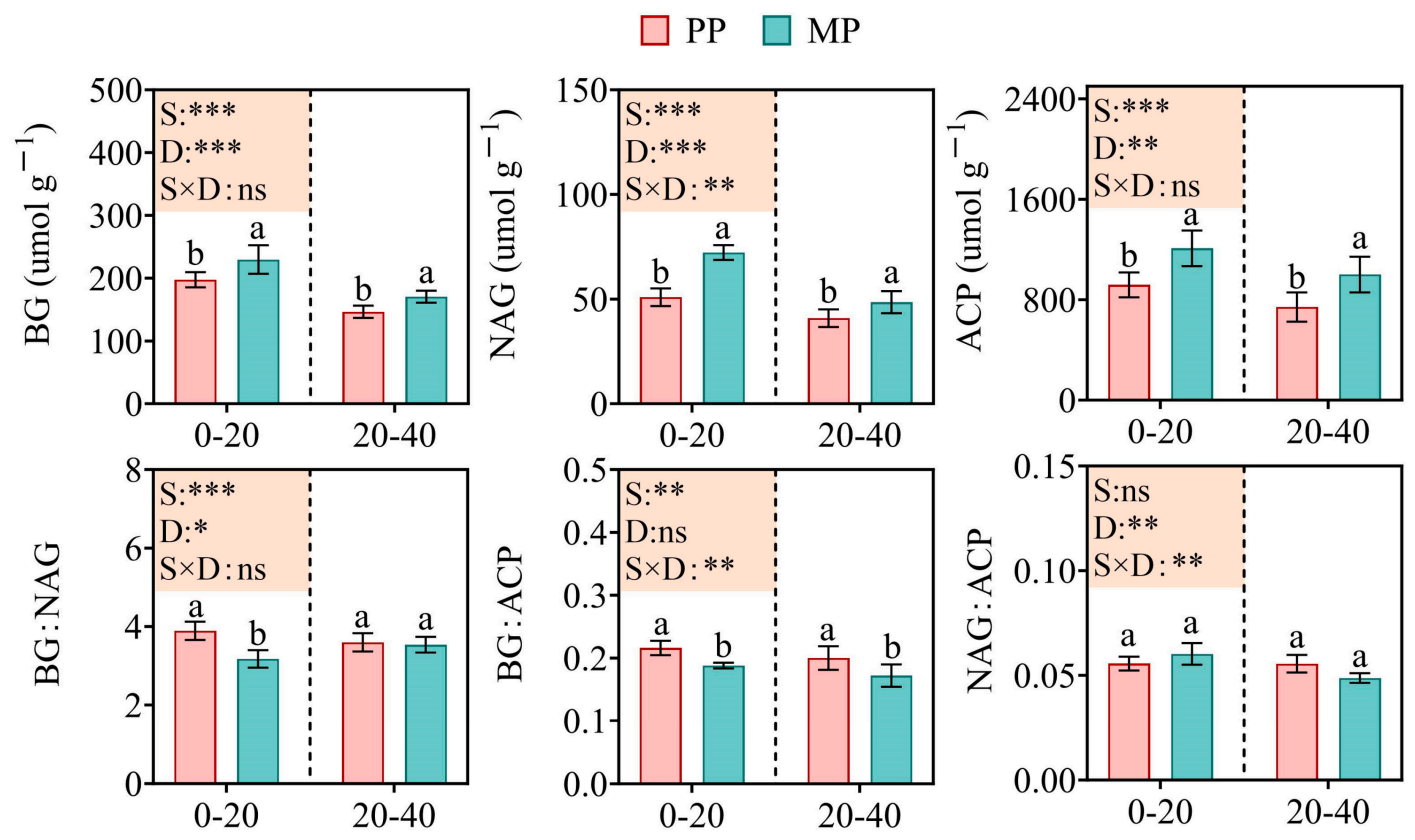
Differences in soil extracellular enzyme activities between PP and MP. Values are presented as means with standard deviations (*n* = 5). Different letters (a, b) above the bars indicate the significant differences (ANOVA, SPSS, *p* < 0.05). PP: Pure Eucalyptus plantation; MP: *Eucalyptus/E. fordii plantation*; BG: β-1,4-glucosidase; NAG: β-N-acetylglucosaminidase; ACP: acid phosphatase. S: stand type; D: soil depth; S × D: interaction effect between soil depth and stand type; *, **, and *** indicate significant differences at *p* < 0.05, *p* < 0.01, and *p* < 0.001, ns indicate *p* > 0.05.

At the overall level, except for the non-significant effect of soil depth on the F:B ratio (Figure 4), both stand type and soil depth significantly influenced soil microbial community structure and MBC, while their interaction only significantly affected Fungi and G+ (*p* < 0.05). Notably, within the same soil layer, soil Fungi, F:B, G+, G- and MBC in MP were significantly higher than those in PP, whereas no significant difference was observed in Bacteria (*p* > 0.05). Both stand type and soil depth significantly affected soil extracellular enzyme activities, while their interaction only significantly affected NAG activity, with no significant effects on BG or ACP activities (*p* > 0.05) (Figure 5). Within the same soil layer, BG, NAG and ACP activities in MP were significantly higher than those in PP. Stand type significantly affected BG:NAG and BG:ACP ratios, but not NAG:ACP; soil depth significantly affected BG:NAG and NAG:ACP ratios, but not BG:ACP; and their interaction significantly affected BG:ACP and NAG:ACP ratios, but not BG:NAG. Notably, in the 0–20 cm layer, no significant difference in NAG:ACP was observed between MP and PP (*p* > 0.05); in the 20–40 cm layer, no significant difference in BG:NAG was observed between MP and PP, while for the other ratios, MP exhibited lower values than PP.

### 2.4. Correlation Analysis Between SOC Fractions and Soil Physicochemical Properties, Soil Microorganisms, and Extracellular Enzymes

**Figure 6:**
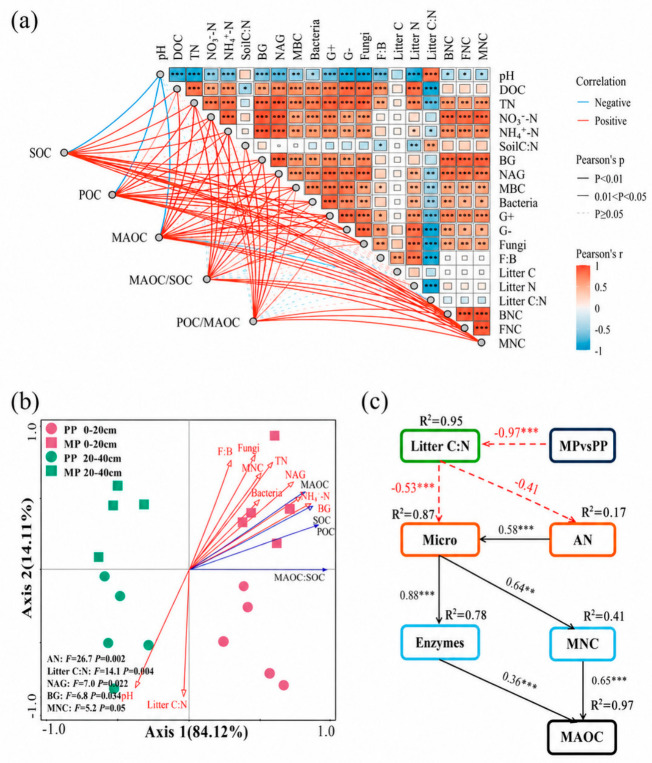
(**a**) Pearson correlation heatmap. (**b**) Redundancy analysis (RDA) of soil microbial community structure, physicochemical properties, and SOC fractions. (**c**) Path diagram illustrating the effects of mixed planting with N-fixing trees on SOC accumulation and stabilization. (*, **, and *** indicate significant differences at *p* < 0.05, *p* < 0.01, and *p* < 0.001); SOC: soil organic carbon; POC: particulate organic carbon; MAOC: mineral-associated organic carbon; MAOC:SOC: proportion of mineral-associated organic carbon in SOC; TN: total nitrogen; NO_3_^−^-N: nitrate nitrogen; NH_4_^+^-N: ammonium nitrogen; AP: available phosphorus; F:B: fungal-to-bacterial biomass ratio; BG: β-1,4-glucosidase; NAG: β-N-acetylglucosaminidase; ACP: acid phosphatase; MBC: microbial biomass carbon; Fungi: fungal biomass; Bacteria: bacterial biomass; G+: Gram-positive bacteria; G-: Gram-negative bacteria; MNC: microbial-derived carbon; FNC: fungal -derived carbon; BNC: bacterial-derived carbon; Litter C: litter organic carbon; Litter N: litter nitrogen; Litter C:N: litter carbon-to-nitrogen ratio.

According to the Pearson correlation heatmap (Figure 6a), SOC, POC, MAOC and MAOC:SOC were significantly positively correlated with TN, NO_3_^−^-N, NH_4_^+^-N, BG, NAG, MBC, Bacteria, G+, Fungi, MNC, FNC, and BNC (*p* < 0.05). Notably, MAOC was also significantly positively correlated with Litter N, and significantly negatively correlated with pH and Litter C:N (*p* < 0.05), whereas MAOC:SOC showed no significant correlation with these variables (*p* > 0.05). Redundancy analysis (Figure 6b) showed that the first two axes explained 84.12% and 14.11% of the variance in SOC fractions, respectively. Among them, AN (NO_3_^−^-N and NH_4_^+^-N), Litter C:N, NAG, BG, and MNC were the main environmental factors driving the differences in SOC and its fraction contents (Figure 6b). Partial least squares path modeling (PLS-PM, Figure 6c) further revealed the cascading pathways through which mixed planting with N-fixing trees was associated with SOC accumulation and stabilization. The results showed that, on the one hand, mixed planting with N-fixing trees was associated with microbial community restructuring through changes in litter quality (lower C:N ratio) (path coefficient = −0.53, *p* < 0.001); on the other hand, it indirectly strengthened the regulation of microbial communities by increasing soil-available nitrogen content (path coefficient = 0.58, *p* < 0.001). The restructured microbial community then promoted the accumulation of MAOC through two parallel pathways, as follows: first, by upregulating extracellular enzyme activities (path coefficient = 0.88, *p* < 0.001), accelerating the decomposition and transformation of organic matter, thereby indirectly contributing to MAOC formation (path coefficient = 0.36, *p* < 0.001); second, by increasing MNC (path coefficient = 0.64, *p* < 0.01), providing potential precursors associated with MAOC formation (path coefficient = 0.65, *p* < 0.001). Collectively, these pathways constitute a synergistic mechanism of “nitrogen enrichment—microbial restructuring—organic carbon stabilization”, which provides evidence for the potential microbially mediated processes through which mixed planting with N-fixing trees enhances the soil carbon sink function in Eucalyptus plantations.

## 3. Discussion

### 3.1. Mixed Planting with N-Fixing Trees Is Associated with Increased SOC Stocks and Shifts the Carbon Pool Toward a More Stable Fraction

The introduction of the N-fixing tree *E. fordii* into Eucalyptus plantations significantly increased SOC and its physical fractions. Relative to pure *Eucalyptus* stands, mixed stands exhibited higher SOC, MAOC, POC, and DOC concentrations in both the 0–20 cm and 20–40 cm layers. The MAOC:SOC ratio increased while the POC:MAOC ratio decreased (Figure 1). These results confirm our hypothesis that mixing with N-fixing trees not only increases soil carbon stocks but also promotes the shift of the carbon pool toward a more stable form.

SOC accumulation and stabilization involve multiple interacting processes [17]. The litter of N-fixing trees showed a 35.12% increase in nitrogen content and a 25.07% decrease in C:N ratio (Table 1). This high-quality, nitrogen-rich litter input provided a favorable organic substrate to the soil system [21,25]. In addition, N-fixing trees may contribute to belowground carbon and nitrogen inputs through multiple pathways, including potential contributions from biological nitrogen fixation, root exudates, and fine root turnover [6]. The aboveground and belowground resource inputs together increased soil nitrogen availability (Table 2) and alleviated microbial nitrogen limitation [26,27]. Once released from N limitation, microbial carbon metabolism efficiency improved. Microbial decomposition of litter and soil organic matter accelerated, as reflected by increased extracellular enzyme activities. At the same time, more of the assimilated carbon was allocated to biomass synthesis, increasing microbial biomass (Figure 4). Both processes contributed to soil carbon accumulation. More importantly, enhanced microbial metabolic activity accelerated the conversion of POC to DOC, which serves as a direct precursor of MAOC [28,29]. The elevated MAOC:SOC ratio thus likely reflects the combined effects of increased microbial carbon metabolism efficiency and effective mineral surface stabilization [28].

The positive effect of mixed stands on soil carbon fractions was evident in both the surface and subsurface layers, and the interaction between stand type and soil depth was not significant (Figure 1). This consistency across depths suggests that the influence of N-fixing trees extends beyond the surface layer through root activity and DOC transport. The well-developed root system of *E. fordii* may deliver labile carbon and nitrogen directly to deeper soils via rhizodeposition and mycorrhizal networks, while DOC produced in surface layers may leach downward and become adsorbed to minerals in deeper horizons [30]. These results highlight that assessments of carbon sink capacity in plantations must consider deep soil processes, otherwise the carbon sequestration potential of mixed stands may be substantially underestimated [30,31].

### 3.2. Soil Nitrogen Enrichment Drives the Microbial Community Restructuring Toward Greater Fungal Contribution

The increase in soil available nitrogen in mixed stands was associated with changes in microbial community structure [32]. Fungal biomass, Gram-positive bacteria and Gram-negative bacteria were all higher in mixed stands than in pure stands, while total bacterial biomass did not change significantly, resulting in a higher F:B ratio. This indicates that nitrogen enrichment selectively favors certain microbial groups [33]. The stronger response of fungi under nitrogen-rich conditions can be explained on two grounds. First, the hyphal network of fungi gives them an advantage in utilizing heterogeneously distributed resources [34]. Litter and root exudate inputs in mixed stands are patchy, and fungi can bridge resource patches through hyphal extension and connectivity, whereas bacteria are limited by their small size and limited dispersal capacity. Second, the lower soil pH in mixed stands (Table 2) favors acid-tolerant microorganisms. Most fungi have a lower pH optimum than bacteria, and the acidic environment suppresses some bacterial groups while fungi maintain normal metabolism [35]. The pH decline likely originates from H^+^ release during nodule nitrogen fixation and from acid production associated with nitrification [36].

Gram-positive and Gram-negative bacteria increased in parallel but total bacterial biomass did not change. Gram-positive bacteria are typically associated with the decomposition of complex organic compounds such as cellulose and lignin, whereas Gram-negative bacteria prefer labile substrates such as low-molecular-weight organic acids and sugars [37,38]. Their concurrent increase suggests that mixed stands provide a greater diversity of carbon sources, with litter contributing complex organic compounds and root exudates providing labile substrates. The stable total bacterial biomass implies that some bacterial groups may have been suppressed or replaced. For instance, nitrogen-fixing bacteria may have declined due to the higher soil nitrogen availability, but this remains speculative because PLFA analysis provides only functional group-level biomass and cannot resolve genus- or species-level changes.

The increase in the F:B ratio has direct implications for soil carbon stabilization [39], as fungi generally exhibit higher carbon use efficiency and produce more microbial-derived carbon than bacteria [39]. In addition, fungal cell walls contain chitin and melanin, which are chemically recalcitrant, and FNC therefore persists longer in soil than BNC [24]. With the increase in relative fungal contribution, more carbon enters the soil through fungal biomass synthesis and is preserved as fungal residues.

### 3.3. Contributions of Microbial-Derived Carbon and Extracellular Enzyme Activities to MAOC Formation

Microbial-derived carbon is recognized as an important precursor of stable SOC, but field evidence for its contribution to MAOC in mixed plantations with N-fixing trees has been lacking [16]. In this study, MNC, FNC and BNC were all significantly higher in mixed stands than in pure stands (Figure 2), and the PLSPM results supported the central role of MNC in promoting MAOC accumulation (Figure 6c) [40]. The increase in FNC exceeded that of BNC, consistent with the larger increase in fungal biomass (Figure 4). Linear correlation analysis further revealed that MNC, FNC and BNC were all significantly positively correlated with MAOC content (Figure 3), confirming the close association between MNC and MAOC formation. Fungal residues contain chitin and melanin, which confer high chemical recalcitrance and strong mineral binding affinity [24]. Fungal residues therefore persist longer in soil and bind more efficiently to minerals than bacterial residues. The elevated MAOC:SOC ratio in mixed stands reflects both an increase in MNC and a compositional shift toward a greater contribution from FNC. This finding is consistent with the microbial carbon pump concept, in which a fungal-dominated community structure may favor the production of MNC with greater mineral affinity, thereby enhancing MAOC stabilization [16,39].

Extracellular enzyme activities also contributed to MAOC formation [12]. BG, NAG and ACP activities were all higher in mixed stands, and this enhancement accelerated the decomposition of POC and the generation of DOC. The DOC produced by enzymatic breakdown of organic matter is either taken up by microorganisms and eventually converted to MNC or diffuses to mineral surfaces and becomes adsorbed. The positive correlation between DOC and MAOC supports the pathway of DOC adsorption to minerals [19]. The PLSPM model showed that the indirect path coefficient from enzyme activities to MAOC was lower than the direct contribution of MNC, indicating that, within the framework of this study, the statistical contribution of MNC pathways to MAOC formation was stronger than the enzyme-mediated pathway within the PLS-PM framework that does not pass through MNC. However, these two pathways are not mutually exclusive, as enzyme activities are essential for generating the DOC that microbes subsequently convert into MNC.

### 3.4. Implications for Sustainable Management of Eucalyptus Plantations

This study demonstrates that mixing *Eucalyptus* with N-fixing trees reduces Litter C:N ratio, increases soil nitrogen availability, is associated with a shift in microbial community structure toward greater fungal contribution, enhances MNC contribution to MAOC, and ultimately increases SOC storage and stability [6,41]. These findings reveal the microbe-mediated mechanism by which mixed plantations enhance soil carbon sinks and offer a new perspective for carbon-focused plantation management. Regulating microbial community structure through changes in litter quality represents a potential pathway to shift the soil carbon pool from labile to stable forms [16].

The greater contribution of MNC relative to enzyme-mediated decomposition further indicates that microbial recycling is the dominant mechanism for stable SOC formation in mixed stands with N-fixing trees [42]. This finding has implications for carbon accounting in plantation forests. When evaluating the carbon sink benefit of mixed stands, microbial community composition and MNC accumulation should be considered as key indicators [21,43]. In addition, the positive effect of mixed stands on SOC was equally evident in the subsurface layer, suggesting that root activity of N-fixing trees influences the deep soil carbon pool [5]. Carbon assessments in plantations should therefore consider the full soil profile rather than being restricted to the surface layer [30]. From a practical management perspective, incorporating N-fixing trees into Eucalyptus plantations may reduce the proportion of Eucalyptus individuals and potentially influence short-term timber production. However, the improvement in soil fertility, carbon stabilization, and long-term site sustainability may provide additional ecological benefits that support sustainable plantation management. Future studies should evaluate the balance between timber yield, carbon sequestration, and soil quality under different mixing ratios. Together, these findings provide a theoretical basis for optimizing plantation management for carbon sequestration and offer a practical pathway toward sustainable plantation production.

Our study provides evidence that mixing Eucalyptus with N-fixing *E. fordii* enhances soil N availability and MAOC accumulation, likely through increased N inputs via BNF. However, BNF activity was not directly quantified in this study, as no artificial inoculation was applied and N fixation relied on indigenous rhizobia. Therefore, the observed N enrichment should be interpreted as indirect evidence consistent with BNF enhancement, rather than direct proof of BNF rates. Unlike mineral N fertilization, which can increase soil N availability but lacks the continuous litter inputs, root-derived carbon inputs, and modification of microbial habitats provided by N-fixing trees, the ecological benefits of mixed plantations extend beyond simple N addition. From a practical management perspective, incorporating N-fixing trees into Eucalyptus plantations may reduce the proportion of Eucalyptus individuals and potentially influence short-term timber production. However, the improvement in soil fertility, carbon stabilization, and long-term site sustainability may provide additional ecological benefits that support sustainable plantation management. Future studies should evaluate the balance between timber yield, carbon sequestration, and soil quality under different mixing ratios, and should consider the full soil profile rather than being restricted to the surface layer. Together, these findings provide a theoretical basis for optimizing plantation management for carbon sequestration and offer a practical pathway toward sustainable plantation production.

## 4. Materials and Methods

### 4.1. Experimental Design

The study site was located at the Tropical Forestry Experimental Center of the Chinese Academy of Forestry Sciences in Pingxiang City, Guangxi Province (21°57′47x–22°19′27″ N, 106°39′50″–106°59′30″ E). The region experiences a subtropical monsoon climate with abundant precipitation concentrated during the rainy season and warm annual temperatures. The dominant soil type is acidic lateritic red soil, and the vegetation belongs to the subtropical monsoon evergreen broad-leaved forest zone [6,44]. Plots with comparable site conditions (elevation, slope aspect, slope position, slope gradient, surface soil, and soil thickness) were selected for the experiment. Three-month-old *Eucalyptus grandis* × *Eucalyptus urophylla* seedlings and 12-month-old *E. fordii* seedlings were planted using the hole-planting method, with hole dimensions of 40 cm × 40 cm × 30 cm, spacing of 3.0 m × 2.0 m, and a planting density of 1667 trees ha^−1^. The experimental plantation was established in January 2019. Five plots of pure Eucalyptus plantation (PP) and five plots of mixed *Eucalyptus* and *E. fordii* plantation (MP) with a 2:1 mixing ratio were established. Each replicate plot measured 20 m × 20 m, and a 40 m buffer zone was set between adjacent plots.

### 4.2. Sample Collection of Litter and Soil

Soil sampling was conducted in March 2024. A five-point sampling method was adopted, as follows: five sampling points were arranged along the two diagonals of each replicate plot, and the five samples were uniformly mixed into one composite sample. Soil samples were collected from the 0–20 cm and 20–40 cm layers using a soil auger with a diameter of 4.5 cm. The collected soils were placed in a 4 °C incubator and transported to the laboratory. After removing stones, roots, litter, and other debris, the soils were passed through a 2 mm sieve. One portion of the soil was stored in a refrigerator at 4 °C for analysis of DOC, MBC, phospholipid fatty acids (PLFAs), NH_4_^+^-N, NO_3_^−^-N, and enzyme activities. Another portion was air-dried for analysis of SOC, POC, MAOC, TN, TP, and available phosphorus (AP). Litter samples were collected from the same five sampling points within each replicate plot. The undecomposed litter layer on the forest floor was carefully collected, pooled into one composite sample per plot, and transported to the laboratory. Litter samples were oven-dried at 80 °C to constant weight, ground, and passed through a 0.15 mm sieve for determination of litter organic carbon and TN contents.

### 4.3. Soil and Litter Physicochemical Analysis

Twenty grams of fresh soil per sample were weighed and dried at 105 °C for two days to determine soil moisture content. Soil pH was measured using a pH meter (FE28-Standard, Mettler, Greifensee, Switzerland) at a soil-to-water ratio of 1:2.5. Soil total nitrogen (TN) and total phosphorus (TP) were determined after H_2_SO_4_–HClO_4_ double-acid digestion. TP was measured using the molybdenum–antimony colorimetric method [45], whereas TN was determined using a continuous-flow chemical analyzer (AA3, Seal Analytical, Hamburg, Germany) [45]. Ammonium nitrogen (NH_4_^+^-N) and nitrate nitrogen (NO_3_^−^-N) were extracted from fresh soil with 2 M KCl and measured using the same continuous-flow chemical analyzer. Available phosphorus (AP) was determined by extracting 2.5 g of soil with 25 mL of 0.045 M HCl–0.03 M NH_4_F solution [46]. Litter C and N concentrations were determined using an elemental combustion analyzer (Elementar Vario Max, Langenselbold, Hesse, Germany).

### 4.4. SOC and Its Fractions

SOC fractions were separated by the wet-sieving method based on particle size [47,48]. Ten grams of air-dried soil (passed through a 2 mm sieve) were placed in a 50 mL centrifuge tube, mixed with 30 mL of 0.5% sodium hexametaphosphate, and shaken for 18 h to disrupt all aggregates. The slurry was rinsed with deionized water on a 53 µm sieve. The fraction passing through the sieve was defined as MAOC (<53 µm fraction), while the fraction retained on the sieve was defined as POC (>53 µm fraction). The POC and MAOC fractions were dried at 60 °C to constant weight. After thorough grinding, both fractions were wrapped in tin foil and measured using a TOC analyzer (MULTIN/C3100, Analytik Jena, Jena, Germany). SOC was also determined using the same TOC analyzer. DOC was extracted with 0.5 M K_2_SO_4_ solution and determined using the wet oxidation method according to the method of Mebius (1960). Fresh soil samples were extracted using a soil-to-extractant ratio of 1:5 (*w*/*v*), and the extracts were filtered before analysis.

### 4.5. Soil Microbial Biomass, Extracellular Enzyme Activities, and Microbial-Derived Carbon

MBC was determined by the chloroform fumigation method [49,50]. Five grams of fresh soil were placed in a 100 mL plastic bottle, fumigated with chloroform for 24 h, and then extracted with 20 mL of potassium sulfate solution by shaking at 200 r/min for 30 min. Non-fumigated soil was extracted in the same manner as a control. The carbon content in the extracts was measured using a TOC analyzer (MULTIN/C3100, Analytik Jena, Germany). MBC was calculated as the difference between fumigated and non-fumigated samples. Bacterial and fungal biomass were determined by PLFA analysis [6]. Four grams of fresh soil were placed in a 50 mL centrifuge tube, and phospholipids were extracted with trichloromethane, separated on a silica gel column to obtain PLFAs, and then esterified with methanol. The resulting fatty acid methyl esters were analyzed on a gas chromatograph (Agilent Technologies, Santa Clara, CA, USA) equipped with the MIDI software system. Soil extracellular enzyme activities including β-1,4-glucosidase (BG), β-N-acetylglucosaminidase (NAG), and acid phosphatase (ACP) were measured using the fluorometric microplate method [51]. One gram of fresh soil was placed in a 150 mL plastic bottle, mixed with 125 mL of sodium acetate buffer, and vortexed for 1 min. Two hundred microliters of soil suspension and 50 μL of enzyme substrate were added to a black 96-well microplate and incubated for 4 h. Fluorescence was measured using a microplate reader (INFINITE M200 PRO, Tecan, Männedorf, Switzerland) at excitation 365 nm and emission 450 nm.

Amino sugars are key components of microbial cell walls, mainly including glucosamine (GluN), galactosamine (GalN), and muramic acid (MurA). Their contents are widely used as biomarkers to indicate the accumulation of MNC in soil, allowing estimation of FNC, BNC, and MNC [52]. In this study, amino sugar contents were determined according to the modified method of Hu [53]. Derivatized amino sugars were analyzed using a gas chromatograph (Agilent 7890B, Agilent Technologies, Santa Clara, CA, USA) equipped with a DB-1 capillary column (30 m × 0.32 mm × 0.25 μm, Agilent Technologies, Santa Clara, CA, USA). BNC and FNC were calculated by the following two formulas, respectively [16].

(1) BNC=MurA×45

(2) $$FNC=\left(GluN/179.2-2\times MurA/251.2\right)\times 179.2\times 9$$

### 4.6. Statistical Analysis

Data were organized using Microsoft Excel software. Differences between PP and MP were evaluated using Student’s t-test. Two-way ANOVA was conducted to examine the effects of stand type, soil depth, and their interaction. Duncan’s multiple range test was used only when comparisons involved more than two groups. Bar charts, linear regression prediction analysis, and associated graphs were generated with Origin 2024 software. Pearson correlation analysis and correlation heatmaps were conducted and plotted using R software. Redundancy analysis (RDA) and its diagram were performed using Canoco 5 software. All data in the figures and tables are presented as mean ± standard deviation (*n* = 5).

## 5. Conclusions

This study provides field-based evidence that introducing the N-fixing tree *E. fordii* into Eucalyptus plantations increases SOC and promotes greater allocation toward MAOC, thereby potentially enhancing long-term carbon stability. The potential mechanism involves a microbial-mediated cascade, as follows:: low Litter C:N from the N-fixing tree increases soil N availability and alters microbial community structure toward greater fungal contribution. This restructuring boosts FNC accumulation and may contribute to MAOC formation. Our work moves beyond descriptive carbon accounting by suggesting a potential microbe-mediated pathway for stable SOC formation in mixed plantations. We conclude that mixing Eucalyptus plantations with N-fixing trees represents a promising strategy to enhance soil carbon sink capacity through microbially mediated processes. Future plantation management for carbon sequestration should consider full-profile soil assessment, using microbial community composition and MNC as early indicators of SOC stability. These findings supported our hypotheses that N-fixing tree integration enhances SOC stabilization through improved litter quality, microbial restructuring, and MNC accumulation.

## Figures and Tables

**Table 1:** Differences in litter organic carbon and nitrogen between PP and MP. The numbers in the table represent the mean ± standard deviation n=5 of each variable. Lowercase letters indicate different levels of significance between PP and MP. PP: Pure Eucalyptus plantation; MP: Eucalyptus/*E. fordii* plantation.

	Litter C (g kg^−1^)	Litter N (g kg^−1^)	Litter C:N
PP	459.16 ± 6.07 a	10.14 ± 0.33 b	45.32 ± 1.60 a
MP	464.92 ± 7.61 a	13.70 ± 0.35 a	33.96 ± 1.29 b

**Table 2:** Differences in soil physicochemical properties between PP and MP. The numbers in the table represent the mean ± standard deviation n=5 of each variable. Lowercase letters indicate different levels of significance between 0–20 and 20–40 soil depth (*p* < 0.05), respectively. The F values are obtained from two-way analysis of variance (*, **, and *** indicate significant differences at *p* < 0.05, *p* < 0.01, and *p* < 0.001). PP: Pure Eucalyptus plantation; MP: *Eucalyptus*/*Erythrophleum fordii plantation*; TN: Total soil nitrogen; NO_3_^−^-N: Soil nitrate nitrogen; NH_4_^+^-N: Soil ammonium nitrogen; TP: Total soil phosphorus; AP: Available soil phosphorus; C:N: SOC to TN ratio.

		pH	TN (g kg^−1^)	NO_3_^−^-N (mg kg^−1^)	NH_4_^+^-N (mg kg^−1^)	TP (g kg^−1^)	AP (mg kg^−1^)	Soil C:N
0–20 cm	PP	4.66 ± 0.21 a	0.98 ± 0.09 b	3.86 ± 0.56 b	9.95 ± 1.78 a	0.42 ± 0.05 a	8.76 ± 1.05 a	14.23 ± 1.07 a
	MP	4.17 ± 0.10 b	1.46 ± 0.14 a	5.64 ± 0.65 a	12.30 ± 1.60 a	0.41 ± 0.07 a	10.81 ± 1.70 a	11.64 ± 0.98 b
20–40 cm	PP	4.84 ± 0.12 a	0.79 ± 0.06 b	2.08 ± 0.52 a	7.35 ± 1.02 a	0.43 ± 0.05 a	9.05 ± 1.48 a	11.61 ± 1.47 a
	MP	4.37 ± 0.07 b	1.08 ± 0.09 a	2.29 ± 0.49 a	8.81 ± 1.48 a	0.41 ± 0.06 a	10.83 ± 2.07 a	10.50 ± 0.58 a
F-value of the stand type (S)		63.67 ***	73.76 ***	15.98 **	8.11 *	0.13	7.00 *	14.81 ***
F-value of the soil depth (D)		10.38 **	40.84 ***	106.49 ***	20.79 ***	0.04	0.05	15.35 ***
F-value of the S × D		0.07	4.26	9.93 **	0.45	0.04	0.04	2.36

## Data Availability

No new data were created or analyzed in this study. Data sharing is not applicable to this article.
